# Feasibility of a New Cuffless Device for Ambulatory Blood Pressure Measurement in Patients With Hypertension: Mixed Methods Study

**DOI:** 10.2196/11164

**Published:** 2019-06-19

**Authors:** Paula AM Ogink, Jelske M de Jong, Mats Koeneman, Mariska Weenk, Lucien JLPG Engelen, Harry van Goor, Tom H van de Belt, Sebastian JH Bredie

**Affiliations:** 1 Department of Internal Medicine Radboud University Medical Center Nijmegen Netherlands; 2 REshape Innovation Center Radboud University Medical Center Nijmegen Netherlands; 3 Department of Surgery Radboud University Medical Center Nijmegen Netherlands

**Keywords:** ambulatory blood pressure monitoring, home blood pressure monitoring, cuffless blood pressure device, hypertension

## Abstract

**Background:**

Frequent home blood pressure (BP) measurements result in a better estimation of the true BP. However, traditional cuff-based BP measurements are troublesome for patients.

**Objective:**

This study aimed to evaluate the feasibility of a cuffless device for ambulatory systolic blood pressure (SBP) measurement.

**Methods:**

This was a mixed method feasibility study in patients with hypertension. Performance of ambulatory SBPs with the device was analyzed quantitatively by intrauser reproducibility and comparability to a classic home BP monitor. Correct use by the patients was checked with video, and user-friendliness was assessed using a validated questionnaire, the System Usability Scale (SUS). Patient experiences were assessed using qualitative interviews.

**Results:**

A total of 1020 SBP measurements were performed using the Checkme monitor in 11 patients with hypertension. Duplicate SBPs showed a high intrauser correlation (*R*=0.86, *P*<.001). SBPs measured by the Checkme monitor did not correlate well with those of the different home monitors (*R*=0.47, *P*=.007). However, the mean SBPs measured by the Checkme and home monitors over the 3-week follow-up were strongly correlated (*R*=0.75, *P*=.008). In addition, 36.4% (n=4) of the participants performed the Checkme measurements without any mistakes. The mean SUS score was 86.4 (SD 8.3). The most important facilitator was the ease of using the Checkme monitor. Most important barriers included the absence of diastolic BP and the incidental difficulties in obtaining an SBP result.

**Conclusions:**

Given the good intrauser reproducibility, user-friendliness, and patient experience, all of which facilitate patients to perform frequent measurements, cuffless BP monitoring may change the way patients measure their BP at home in the context of ambulant hypertension management.

## Introduction

An elevated blood pressure (BP) is a major risk factor for cardiovascular morbidity and mortality [1]. BP is, however, a highly variable vital parameter, and circumstances under which measuring takes place may influence the result extensively [2-4]. Compared to office BP measurement, home BP measurement predicts cardiovascular risk better [5-8]. The predictive value increases progressively with the number of home measurements [9]. Thus, to improve assessment of BP for diagnosis and management of high BP, the BP needs to be monitored frequently, preferably at home.

For patients with hypertension, home blood pressure monitoring (HBPM) is easy to perform, reliable, and reproducible [10]. Therefore, it is recommended as a routine component of BP monitoring in the American Society of Hypertension and the American Heart Association guidelines [5]. The use of HBPM improves hypertension control and the associated outcomes [11-14]. At home, BP is predominantly measured with an oscillometric BP monitor, which uses an arm cuff. In daily practice, patients use different types of BP monitors, which, in most cases, have been validated according to the international standard and have been checked by their provider [15]. However, in a cross-sectional study by Ruzicka et al, 30% of home BP monitors were found to be inaccurate with use of a stringent criterion of 5 mm Hg difference between measurements. Using a different threshold for accuracy (difference of more than 10 mm Hg), 16 of 210 (8%) HBP monitors were inaccurate for systolic BP (SBP) and 18 (9%) were inaccurate for diastolic BP [16]. Although an automatic BP monitor is relatively easy to use and inexpensive [5], measuring BP is time-consuming and may be perceived as inconvenient. In addition, various factors may influence the accuracy of measuring BP, such as discomfort by inflation of the cuff [17] and inappropriate cuff size and cuff position at the arm in relation to the heart level [18].

A new technique has been developed to measure SBP fast and easy without the use of a cuff, which is applied in new devices such as the Checkme Pro Health Monitor (Shenzhen Viatom, China). An algorithm calculates the SBP based on the pulse transit time determined by the peripheral capillary oxygen saturation (SpO_2_) measurement (an estimate of the amount of oxygen in the blood and is the percentage of oxygenated hemoglobin compared to the total amount of hemoglobin in the blood), the electrical electrocardiogram (ECG) signal, and the individual’s arterial compliance [19]. For the latter, the cuffless device needs a calibration procedure, which is developed by entering a classically obtained SBP. Calibration needs to be repeated monthly. An SBP measurement with the Checkme monitor takes less than 30 seconds, which could increase the willingness of patients to measure their SBP more frequently. Cuffless BP measurement is an emerging technique, which may lead to an increased patient compliance in measuring BP at home and promoting a larger number of BP results for hypertension management. Moreover, the Checkme monitor captures a single-lead ECG and photoplethysmogram signal in the same 30 seconds.

Recently, we evaluated the validity of the Checkme monitor’s SBP results by using criteria of the European Society of Hypertension for validating new BP devices [20]. This validation protocol may be considered inadequate, as it lacks consensus about the quality of the BP measurement to be used for calibrating cuffless devices. However, results obtained with the Checkme monitor were promising over a wide range of BP levels [20]. A recent study showed promising results of vital parameter measurements in an inpatient setting [21]. Since the previous study by Schoot et al was performed under demanded controlled circumstances, there was a need to evaluate the performance of the Checkme monitor in an uncontrolled home setting.

To assess feasibility of the Checkme monitor in an outpatient setting, we studied the performance, user-friendliness, and patient experience of the Checkme me in participants’ home settings.

## Methods

### Research Design, Setting, and Participants

We conducted a pilot study using a mixed method approach. To determine performance of the SBP measurement, we systematically assessed the reproducibility of the Checkme monitor, its comparability to a home BP monitor, and the performance of daily vital measurements with the Checkme monitor using video analysis. A System Usability Scale (SUS) questionnaire was used to determine the user-friendliness. Patient experience was assessed using a semistructured interview following the Unified Theory of Acceptance and Use of Technology 2 (UTAUT2) framework. Participants were recruited from the hypertension outpatient clinic of an academic hospital in The Netherlands from April 2017 to May 2017. The institutional review board approved the study (ID: 2017-3241). All participants provided signed informed consent after written and verbal information were obtained.

### Inclusion and Exclusion Criteria

Patients were considered eligible if they were receiving medical treatment for high BP, accustomed to home BP measurements with their own blood pressure monitor, of age ≥ 18 years, and had the cognitive ability to understand instruction and perform measurements correctly after instruction. Patients with a pacemaker and pregnant women were excluded.

### The Checkme Pro Health Monitor

**Figure 1:**
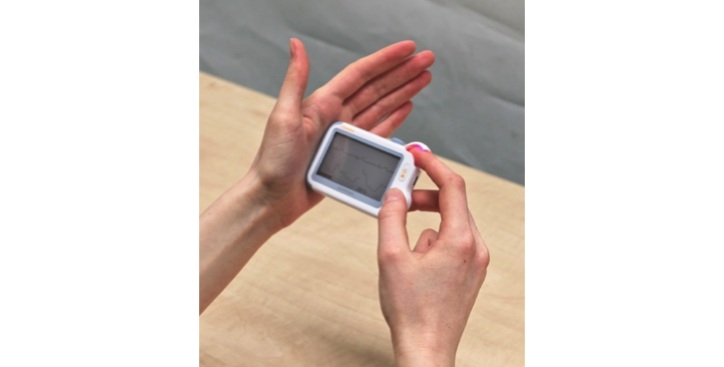
Demonstration of a systolic blood pressure measurement using the Checkme Pro Health Monitor. The right thumb, middle finger, and left palm are placed on the electrocardiogram sensors. The right index finger is placed on the built-in SpO_2_ (peripheral capillary oxygen saturation) sensor. Systolic blood pressure measurement is performed in less than 30 seconds, holding the device steady at heart level.

We evaluated the Checkme Pro Health Monitor, which measures SBP without the use of a cuff. The device also measures a one-lead ECG, heart rate, and SpO_2_ in one measurement called “daily check.” The method of measurement of these vitals by the Checkme monitor is shown in Figure 1. The right thumb, right middle finger, and left palm are placed on the ECG sensors. The right index finger is placed on the built-in SpO_2_ sensor. To increase accuracy of the results, the device needs to be held steady at the heart level during the measurement. The latest version of the Checkme monitor, used in this study, is cleared by the US Food and Drug Administration (FDA) for measuring these vitals (FDA 510k release: K150869; Device Name: CheckMe Pro Health Monitor; Regulation Number: 21 CFR 870.2300; Regulation Name: Cardiac Monitor Including Cardiotachometer and Rate Alarm; Regulatory Class: Class II; Product Code: MWI on November 6, 2015) and complies with the Conformité Européene (CE) marking medical devices directive (CE certificate was issued on behalf of TüV Rheinland LGA Products GmbH notified body [CE 0197] on Viatom’s Health Monitor models “Checkme Pro, Plus, Pod, and Lite” [standard MDD 93/42/EEC, Annex II; Certificate HD60107767 0001; April 27, 2016]).

### Study Procedures

**Figure 2:**
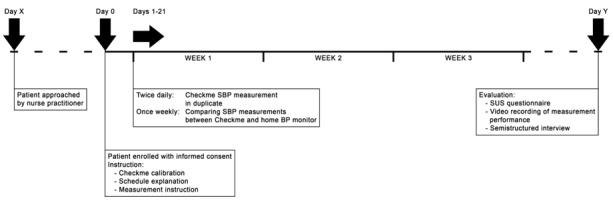
Timeline of the study procedures. Dotted lines represent a variable time of 0-5 days between day X and day 0, and 0-5 days between the end of the study period and day Y. BP: blood pressure; SBP: systolic blood pressure; SUS: System Usability Scale.

The study timeline and procedures are shown in Figure 2. Two trained researchers instructed the participants on the study procedures and how to perform the SBP measurement with the Checkme monitor, and checked the way they performed their regular home SBP measurement using their own home BP monitor. Before the start of the study, the Checkme monitor was calibrated for SBP measurement according to manufacturer’s instructions. To determine a reference SBP to calibrate the Checkme monitor, a standard duplicate BP measurement was performed using a validated automatic BP monitor at the outpatient clinic (Vital Signs Monitor 300 series, Welch Allyn, Skaneateles Falls, NY) after an initial 5 minutes of rest. The participants performed the measurements with the Checkme monitor in duplicate twice daily, in the morning and evening, for a period of 3 weeks. They were instructed to perform the second measurement immediately after the first measurement under the same circumstances.

In addition, the participants performed regular BP measurement once weekly with their own home BP device. Participants were asked to perform one duplicate SBP measurement with the Checkme monitor and one duplicate home BP measurement using their own conventional BP monitor, in a random order.

After 3 weeks, the correct use of the Checkme monitor by the patient was checked with a video recording of the SBP measurement. The user-friendliness was assessed using the SUS questionnaire, and the patient’s experience was determined with a semistructured interview.

### Analysis of Reproducibility

To obtain the intrauser reproducibility of a duplicate SBP measurement, two values of one duplicate measurement were correlated and the level of variation was categorized as <5 mm Hg, <10 mm Hg, and >15 mm Hg. Both the Checkme and home BP monitors were tested for reproducibility. The paired SBP measurements with the Checkme monitor and home BP monitor were correlated to obtain comparability. The mean difference and level of variation in SBP measurements between the Checkme and home BP monitors were calculated. In addition to the paired measurements, the means of all SBP measurements with the Checkme and home BP monitors were correlated. For each participant, all SBP values measured with the Checkme monitor, their home BP monitor, and the hospital monitor were plotted in a diagram to show the variation of SBP over time measured with different devices.

### Analysis of the Correct Use of Checkme by the Patient

Two researchers independently assessed the use of Checkme by the patient, by checking the video-recorded measurements with a scoring sheet based on the principles of Gelbart et al [22] and Van Der Heide et al [23]. Thirteen steps were distinguished for the SBP measurement with the Checkme monitor. All items for the use of the Checkme monitor were categorized as “badly performed” or “not done” (0 points), “suboptimal” or “too late” (1 point), and “perfectly done” (2 points). Findings were compared and discussed until a consensus was reached.

### Analysis of Patient Experience

**Figure 3:**
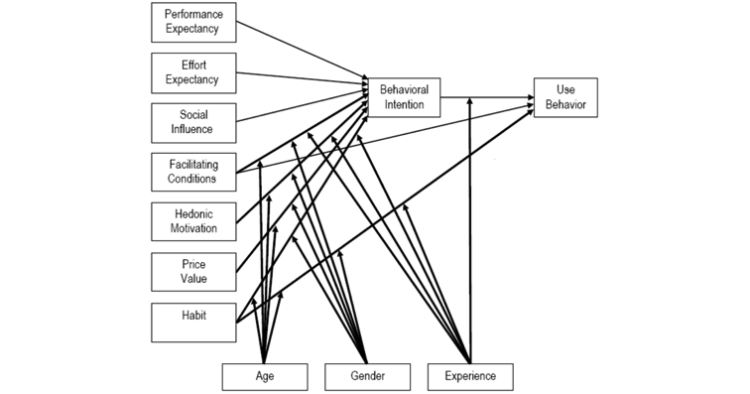
Interview framework of the Unified Theory of Acceptance and Use of Technology 2.

The semistructured interviews following the UTAUT2 framework [24] (Figure 3) were conducted in Dutch with the participants. The UTAUT2 framework consists of four major themes: performance expectancy, effort expectancy, social influence, and facilitating conditions. Performance expectancy is defined as the degree to which an individual believes that using the Checkme monitor will help him/her. Effort expectancy is defined as the degree of ease associated with the use of the Checkme monitor. Social influence is defined as the degree to which an individual perceives that significant others believe he/she should use Checkme. Facilitating conditions are defined as the degree to which an individual believes that an organizational and technical infrastructure exists to support the use of the Checkme monitor [25].

Interviews were audio recorded and transcribed verbatim using qualitative data analysis software (ATLAS.ti 7.1, Scientific Software Development GmbH, Berlin, Germany). Transcripts were independently analyzed by two investigators to identify barriers, facilitators, and positive and negative effects of the use of the Checkme monitor. Findings were discussed until a consensus was achieved. The barriers and facilitators were rewritten into general statements and subdivided according to the themes of the UTAUT2 interview framework. The magnitude of each statement was determined by the number of interviews the statement was mentioned in. A validated Dutch translation [26] of the SUS questionnaire [27] on the usability of the Checkme monitor was used to determine the user-friendliness, scored between 0 and 100, as described by Brooke et al [27]. The interpretation of the SUS score was in accordance with that provided by Bangor et al [28]. A score above 90.9 was considered “best imaginable,” a score above 85.5 was considered “excellent,” a score above 71.4 was considered “good,” a score above 50.9 was considered “sufficient,” and a score below or equal to 50.9 was considered “poor.”

### Statistical Analyses

All statistical analyses were performed using IBM SPSS, version 22.0 (IBM Corp, Armonk, NY). Normally distributed data were presented as mean and SD. Descriptive statistics were presented as median and quartiles in case of nonnormally distributed data. Differences were tested using a *t* test in case of normal distribution of the data and the nonparametric Wilcoxon test in case of nonnormally distributed data. Correlations were calculated with the Spearman rank correlation coefficient.

## Results

### User Statistics

One of 12 participants enrolled in the study was excluded from participation and analysis due to repeated failure of BP calibration. One participant did not own a BP monitor and visited the hospital for weekly BP measurements. Average instruction time was 20-40 minutes. The characteristics of the 11 participants who completed the study period are summarized in Table 1. The SBP readings of the participants’ home BP monitors strongly correlated with those of the automatic hospital BP monitor at baseline (*R*=0.88, *P*<.001). Eleven participants performed a total of 1020 measurements with the Checkme monitor. In 209 measurements (20.4%), the Checkme monitor was not able to measure the SBP. The success rate for SBP measurement varied among participants, with a mean success of 71%, ranging from 42% to 100%.

**Table 1:** Participant characteristics (N=11).

Characteristics		Study population
**Gender, n (%)**		
	Female	4 (36)
	Male	7 (64)
**Ethnicity, n (%)**		
	Caucasian	10 (91)
	Black	1 (9)
Age (years), mean (SD)		57 (11.5)
Systolic BP^a^ (mm Hg), mean (SD)^b^		140.7 (13.7)
Diastolic BP (mm Hg), mean (SD)^b, c^		86.3 (11.0)
Use of BP-lowering medication, n (%)		9 (82)
Use of home monitor, n (%)		10 (91)
**Brand, n^d^**		
	Withings	3
	Microlife	2
	Omron	1
	Beurer	1
	A&D Medical	1
	Medion	1
	Cresta	1
Frequency per month, mean (SD)		6.8 (6.2)

^a^BP: blood pressure.

^b^BP measured by trained investigator with a Welch Allyn Automatic BP monitor at day 0.

^c^Data shown for only 10 patients, because of the lack of diastolic BP data in one patient.

^d^N=10.

### Reproducibility

**Figure 4:**
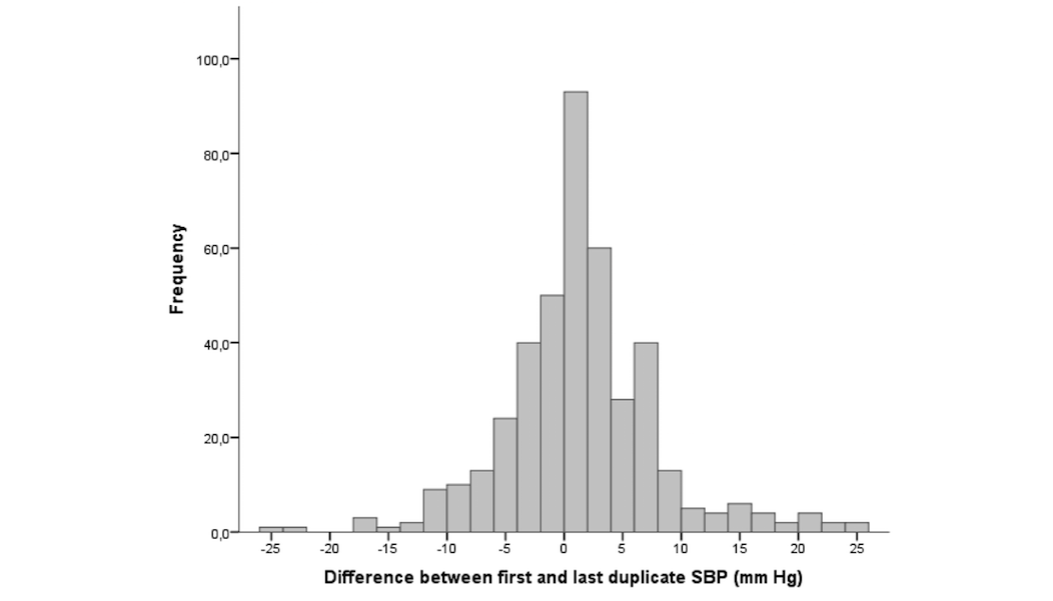
Frequency of the difference within duplicate SBP measurements. SBP: systolic blood pressure.

**Figure 5:**
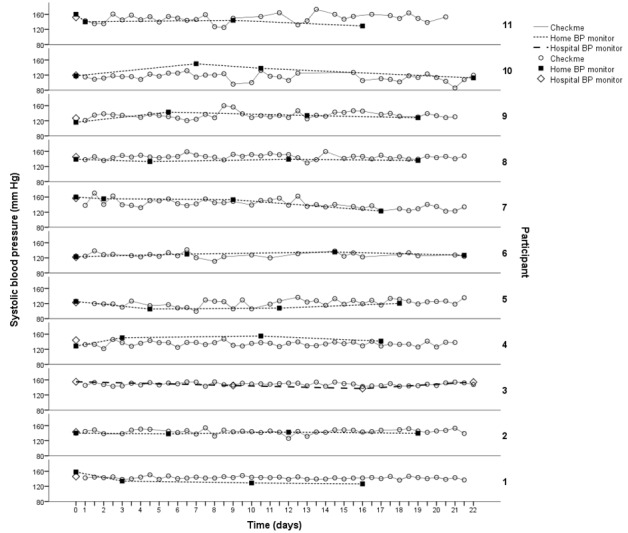
Systolic blood pressure during follow-up for each participant, measured with Checkme and home BP monitor or hospital BP monitor. BP: blood pressure.

The paired results of duplicate SBP measurements of the Checkme monitor correlated well over the whole range of BP levels (*R*=0.86, *P*=.001). Of the 420 complete duplicate SBPs, paired results of 374 (89%) duplicates varied within 10 mm Hg, of which 286 (68% of total) varied within 5 mm Hg. Paired results of 22 (5%) duplicate SBPs varied more than 15 mm Hg. Variations of the paired results of duplicates are shown in Figure 4. Of the 22 duplicates with a difference of more than 15 mm Hg, 11 were obtained from only two participants. The paired results of duplicate SBP measurements with the home BP monitors correlated strongly (*R*=0.91, *P*<.001). Of the 40 complete duplicate SBPs, 38 (95%) varied within 10 mm Hg, of which 27 (67% of total) varied within 5 mm Hg. No measurement exceeded a variation of 15 mm Hg. For each participant, all SBP values measured with the Checkme monitor (twice daily), the home BP monitor (once weekly), and the hospital monitor (once) are plotted in Figure 5.

The measurements of the Checkme and home BP monitors correlated weakly (*R*=0.47, *P*=.007). The mean results of the paired measurements with the Checkme monitor were 0.55 mm Hg (SD 12.32) higher than those of the home BP monitors. Of the 32 paired SBP values, there was a difference between the Checkme and home BP monitors of <5 mm Hg in 7 pairs (22%), <10 mm Hg in 18 pairs (56%), and <15 mm Hg in 26 pairs (81%).

The mean SBP of both devices over 3 weeks correlated strongly (*R*=0.75, *P*=.008). The Checkme monitor had a mean systematic difference of 0.26 mm Hg (SD 7.66) for mean SBP over 3 weeks compared to the home BP monitors. In addition, 36.3% of the mean SBP measurements with the Checkme and home BP monitors varied within 5 mm Hg and 90.9% varied within 10 mm Hg. No measurement exceeded a variation of 15 mm Hg.

### Correct Use of the Checkme Monitor

In total, 36.4% (n=4) of the participants performed the measurement with the Checkme monitor correctly (“well done”) in all 13 items, 54.5% (n=6) performed the measurement with one mistake, and 9.1% (n=1) performed the measurement with two mistakes. No participants made more than two mistakes. The most frequent mistake was not keeping the Checkme monitor at heart level (5/11). During video recording, 1 of the 11 participants did not receive a valid SBP result. The hands of this participant were shaking due to the side effects of the medication, and he did not find support by resting his arms on the table. However, at home, this participant achieved a valid SBP in 80 of 104 measurements (77%).

### Patient Experience

Interviews lasted for 15-35 minutes. All perceived barriers and facilitators could be subdivided into one of the five themes of the UTAUT2 interview framework. Most significant barriers and facilitators are described here, and all barriers and facilitators are summarized in Table 2.

Performance expectancy could be divided into two subsequent topics: the features and possibilities of the Checkme monitor and the measurement results produced by the Checkme monitor. For the possibilities of the Checkme monitor, six participants perceived the inability of Checkme to measure DBP as a barrier. They considered DBP to be as important as SBP. At times, the Checkme monitor did not report a result for SBP, which was a barrier for seven participants. This was sometimes perceived as bothersome, since the cause was unclear and the measurement had to be repeated. Another barrier mentioned by three participants was the occasional big difference in SBP measured with the Checkme monitor compared to the home or hospital BP monitor. This reduced their trust in Checkme’s performance. One participant, on the other hand, reported that the Checkme and home BP monitors correlated well, which increased her trust in the Checkme monitor.

**Table 2:** Barriers and facilitators for use of the Checkme monitor and the number of interviews these were mentioned in, according to the themes of the Unified Theory of Acceptance and Use of Technology 2 interview framework and subsequent topics.

Variable			Barrier	Facilitator
**Performance expectancy**			16	1
	**Possibilities of Checkme**			
		The device measures only SBP^a^	6	0
	**Outcomes of Checkme**			
		At times, the Checkme did not report a result for SBP and/or SpO_2_^b^	7	0
		The big/small difference between the home BP^c^ monitor and the Checkme leads to less/more trust in the Checkme	3	1
**Effort expectancy**			7	22
	**Performing measurements**			
		The Checkme is easy to use	0	11
		With the Checkme, a measurement is quickly performed	1	4
		BP can be measured with the Checkme without the use of an arm cuff	0	3
		Daily check cannot be performed with cold hands	1	0
	**Design of Checkme**			
		The Checkme is small and can be taken everywhere	0	4
		The Checkme does not have a backlight in the touch screen	2	0
		The Checkme is not a standard BP monitor, which decreases trust in results	2	0
		The font size of the results screen is very small	1	0
**Social influence**			0	1
	Measuring BP with the Checkme can be done without any help		0	1

^a^SBP: systolic blood pressure.

^b^SpO_2_: peripheral capillary oxygen saturation.

^c^BP: blood pressure.

Effort expectancy was defined through two topics: performing measurements and the design of the Checkme monitor. For performing measurements, all participants considered the Checkme monitor easy to use and four could quickly perform a measurement. Three participants perceived the Checkme monitor to be a facilitator that can measure SBP without the use of an arm cuff, mostly because the arm cuff on their home BP monitor was uncomfortable. Regarding the design of the Checkme monitor, the most significant facilitator perceived was the small size of the device (n=4).

In addition, three participants thought the Checkme monitor would be unsuitable for the elderly, because of their decreased fine motor skills. Automatic synchronization of results was preferred by eight participants, either to their medical record or an online app, to be able to monitor the results of medication, diet, and physical activities and discuss the results with their doctor. Two other suggestions were increasing the font size and addition of a backlight to the screen.

Five participants wanted to use the Checkme monitor in the future instead of their own home BP monitor. Three other participants wanted to use the Checkme monitor in the future only on certain conditions, for example, if the device reports reliable results. The remaining three participants did not want to use Checkme in the future. Eight participants would recommend the Checkme monitor to other patients. Participants gave the monitor a median score of 7.5 (interquartile range: 5.0-8.0) on a scale of 1 to 10, with individual scores ranging from 1.0 to 9.0. The mean SUS score was 86.4 (SD 8.3) with a range of 72.5-97.5, which indicates high user-friendliness.

## Discussion

### Principal Findings

This study provides new insights about the use, performance, and patient experience of an FDA-approved cuffless BP-measuring device in patients who are used to measuring their blood pressure at home. Adequate intrauser reproducibility of cuffless SBP measurement was observed in the majority of participants, and the Checkme monitor was well adopted in the home setting. Patients indicated an increased willingness to take their BP measurement because of its ease of use. Thus, the large variety of cuff-based BP monitors currently used by patients in home BP monitoring does not necessarily serve as a gold standard to compare new devices for home monitoring. In addition, the easily obtained large number of the Checkme SBP measurements may provide a better picture of the actual BP variation over time, which can be easily missed by taking only one home measurement every week.

The SBP measured by the Checkme monitor was comparable to that measured by an in-hospital reference monitor, with a mean difference of 2.6 (SD 12.1) mm Hg [20]. Other studies compared different cuffless SBP-measuring devices to a reference monitor. Poon et al [29] described a mean difference of 0.6 (SD 9.8) mm Hg, and Boubouchairopoulou et al [30] found a mean difference of 3.2 (SD 6.7) mm Hg, which is, to a great extent, comparable to the results of this study. In addition, this study showed that currently, an unrestricted range of BP monitors from different manufacturers are being used for home BP monitoring. Both the variety of home BP monitors and the uncontrolled use in a home setting may contribute to the observed differences. Although home BP devices should ideally be on the list of validated monitors and the circumstances under which measurements are taken should be standardized, the added value of home BP monitoring is the increasing number of results, not the absolute value of each of them [31]. Robust hypertension management is based on the average of a large series of BP measurements rather than a single clinic measurement [31]. The majority of participants could produce a series of valid measurement results, and most of the unsuccessful SBP measurement attempts were observed in a small number of participants. Failure to produce valid SBP readings may be caused by several factors. Since the cuffless technique requires an ECG and SpO_2_ signal to produce an SBP result, factors influencing ECG and SpO_2_ accuracy may lead to unsuccessful measurements with no SBP results. These factors include poor perfusion (cold fingers) and skin color [32,33]. Performance-related factors such as moving during the measurement or applying too much pressure on the sensors [34] may disturb the SpO_2_ signal and thereby influence the SBP result, which suggests that proper user instructions are necessary. Technical factors such as system failure, incorrect calibration procedure, or imperfections in the algorithm may also influence the BP results.

Another issue of the cuffless BP measurement technique is the need of a classic reference BP measurement to calibrate the calculating algorithm for individual vascular compliance. An international standard for this calibration procedure is still lacking in existing protocols for new BP device validation [35]. Schoot et al [20] recently performed a pragmatic validation study with the Checkme monitor by using standardized measurement conditions, which revealed promising results.

Despite its easy-to-use concept, accurate self-measuring with the Checkme monitor was not completely adequate after a single instruction at the start of this study. This phenomenon was also observed in studies with conventional BP monitors, which reported that 52%-65% of patients missed at least one step of the BP measurement process [36,37]. Milot et al found that only 18% of patients performed the classic BP measurement with cuff with excellence [38], and Wagner et al found that none of the participants performed BP measurement correctly [39]. Compared with these observations in classical cuff-based BP measurement, the correct use of the Checkme monitor in our study was much better. The only observed mistake was not holding the device at the heart level, which has a minor effect on the SBP result [20]. Other mistakes concerning the use of the Checkme monitor were not observed. This is in contrast to the observation during classic BP measurement, in which various other errors, with respect to cuff usage and position, can occur. An important finding of the present study is that user instruction needs attention, both at the start and during long-term use, to increase the quality of BP readings. This may be achieved by optimizing the patient instruction by using the protocol described by Mengden et al as a guide [40] or using video instructions [41].

Performance expectancy and effort expectancy were most mentioned in the interviews, and only a few barriers and facilitators were mentioned on social influence and facilitating conditions. This can be explained by the short follow-up period and the fact that Checkme is a new unknown device. The two most prominent issues of performance expectancy for participants are that the Checkme monitor only measures SBP and not DBP, and the monitor sometimes fails to produce a valid SBP result. The cuffless BP measurement technique is currently unable to determine DBP accurately. However, it is internationally accepted that SBP is the primary target in managing cardiovascular risk in most patient groups, except in elderly people [42]. Some participants also suggested improving the design of the Checkme monitor. Although requirements for medical devices are dictated by appropriate legislative bodies such as the European directive (93/42/EEC, the Medical Devices Directive) for the European devices, five requirements for home monitoring with wearable sensors have been described by Korhonen et al: reliability and durability, looks and unobtrusiveness, user identification, communication, and zero maintenance and fault recovery [43]. Cuff-based home BP monitors meet the first and last requirement. The Checkme monitor formally meets the second, third, and fourth criteria with its size, personal user profiles, and ability to share readings, respectively. This study also provides new information about the reliability through its evaluation of intrauser reproducibility of the Checkme monitor in home monitoring. The memory capacity of the Checkme monitor and the ability to automatically share saved readings bypasses the imprecision of self-reported BP readings by patients, which appeared to range from 0% to 100% in a study on 30 patients with hypertension [44]. Further, it enables physicians to intervene and adjust medication, since patients are often not able to interpret the readings of SBP correctly [45,46].

### Strengths and Limitations

The strength of this pilot study is that SBP was measured by the cuffless Checkme device in a home-based setting. This is the first study in which the feasibility, usability, and acceptability of a cuffless BP measuring device in a home monitoring setting were assessed using a mixed method study design. We obtained a large number of home measurements in the morning and evening, as recommended by the European Society of Hypertension/European Society of Cardiology [47] for well-instructed patients who were involved in self-management. Patient experience and performance were evaluated by a widely used and reliable questionnaire to determine the feasibility of different products, and all interviews followed an interview guide derived from a well-known interview framework [28,48]. A weakness of this study was the small study sample, which may not guarantee complete saturation in all qualitative aspects. The relative short follow-up may also have led to an incomplete user experience. In addition, the fact that medication use may instantly influence measurements could induce device-independent differences. However, the blood pressure results that were compared were based on daily averages or were time-related (measurement with both devices at the same time). To confirm the current results, this study should be repeated on a larger scale. Some adjustments of the methods need to be taken into consideration, including more explicit user instructions and a restricted set of validated home BP monitors as a reference. Future studies should focus on the cause and mechanisms of failures to measure SBP with Checkme in some patients or settings. Furthermore, an international validation protocol for the calibration procedure of a cuffless device is needed. The Institute of Electrical and Electronics Engineers Standard for Wearable Cuffless Blood Pressure Measuring Devices [49] should incorporate a norm for such devices.

### Implications for Practice

It is highly possible that the use of cuffless SBP devices will become part of common practice in hypertension and cardiovascular risk management in the near future. Therefore, health care professionals should be aware of this development and familiarize themselves with the specific characteristics of these devices. They could explore possibilities such as smart data analysis and connectivity with electronic health records. Patients and their relatives should not hesitate to discuss the possibilities in home monitoring with their health care professionals. If they start using these devices, it may provide them with better insight into their health status and recovery, with minimum effort.

### Conclusions

As confidence in BP measurement results continues to increase, and if international consensus on the calibration process is reached, cuffless BP monitoring devices such as the Checkme monitor may change the way patients measure their BP at home, in the context of ambulant hypertension and cardiovascular risk management. A major advantage of the Checkme monitor in addition to the current use of cuff-based BP home monitors is that the former stimulates the patient in taking a larger number of BP readings because of its easy-to-use design.

## Abbreviations

BP: blood pressure
CE: Conformité Européene
DBP: diastolic blood pressure
ECG: electrocardiogram
FDA: Food and Drug Administration
HBPM: home blood pressure monitoring
SBP: systolic blood pressure
SpO_2_: peripheral capillary oxygen saturation
SUS: System Usability Scale
UTAUT2: Unified Theory of Acceptance and Use of Technology 2
